# Using shared goal setting to improve access and equity: a mixed methods study of the Good Goals intervention in children’s occupational therapy

**DOI:** 10.1186/1748-5908-7-76

**Published:** 2012-08-16

**Authors:** Niina Kolehmainen, Graeme MacLennan, Laura Ternent, Edward AS Duncan, Eilidh M Duncan, Stephen B Ryan, Lorna McKee, Jill J Francis

**Affiliations:** 1 Health Services Research Unit, University of Aberdeen, 3rd floor, HSB, Foresterhill, Aberdeen, AB25 2ZD, UK; 2 Health Services Research Unit and Health Economics Research Unit, University of Aberdeen, 3rd floor, HSB, Foresterhill, Aberdeen, AB25 2ZD, UK; 3 Nursing, Midwifery and Allied Health Professions Research Unit, University of Stirling, Stirling, FK9 4LA, UK; 4 Health Services Research Unit and Aberdeen Health Psychology Group, University of Aberdeen, 2nd floor, HSB, Foresterhill, Aberdeen, AB25 2ZD, UK

## Abstract

**Background:**

Access and equity in children’s therapy services may be improved by directing clinicians’ use of resources toward specific goals that are important to patients. A practice-change intervention (titled ‘Good Goals’) was designed to achieve this. This study investigated uptake, adoption, and possible effects of that intervention in children’s occupational therapy services.

**Methods:**

Mixed methods case studies (n = 3 services, including 46 therapists and 558 children) were conducted. The intervention was delivered over 25 weeks through face-to-face training, team workbooks, and ‘tools for change’. Data were collected before, during, and after the intervention on a range of factors using interviews, a focus group, case note analysis, routine data, document analysis, and researchers’ observations.

**Results:**

Factors related to uptake and adoptions were: mode of intervention delivery, competing demands on therapists’ time, and leadership by service manager. Service managers and therapists reported that the intervention: helped therapists establish a shared rationale for clinical decisions; increased clarity in service provision; and improved interactions with families and schools. During the study period, therapists’ behaviours changed: identifying goals, odds ratio 2.4 (95% CI 1.5 to 3.8); agreeing goals, 3.5 (2.4 to 5.1); evaluating progress, 2.0 (1.1 to 3.5). Children’s LoT decreased by two months [95% CI −8 to +4 months] across the services. Cost per therapist trained ranged from £1,003 to £1,277, depending upon service size and therapists’ salary bands.

**Conclusions:**

Good Goals is a promising quality improvement intervention that can be delivered and adopted in practice and may have benefits. Further research is required to evaluate its: (i) impact on patient outcomes, effectiveness, cost-effectiveness, and (ii) transferability to other clinical contexts.

## Introduction

Around 17% to 19% of all children have a long-term health condition; 8% of these are severe (*e.g.*, autism, cerebral palsy). Most of these children receive input from therapy services ( *e.g.*, occupational therapy, physiotherapy) at some point of their childhood. The organisation and delivery of children’s services varies widely, however the literature indicates that the challenges they face are similar across contexts. Lack and inequity of access are the main challenges in children’s therapy services internationally and across professional boundaries [1-8]. Children wait up to 12 months for an initial appointment and longer for treatment. These problems are at the core of families’ dissatisfaction with healthcare [4,8,9]. Lack of access is associated with family distress [8,9] and psychosocial problems for the child [5], and the delays in initiation of treatment represent a lost opportunity to prevent problems in the child’s development, achievement, and quality of life [10,11]. Previous research has indicated that the access and equity problems are unlikely to be resolved just by increasing resources [1], and with increasing pressure on the healthcare services to reduce costs, finding more efficient ways of working is a priority. Yet, there is currently little evidence to guide services on how to improve practice [12].

Research evidence from different fields indicates that: service access, equity, and efficiency are related to clinicians' actions at assessment, treatment, and discharge; these actions mediate the effects of organisational and patient characteristics; and that increasing resources is unlikely to resolve service delivery problems because clinicians do not always use resources well [1,13-20]. Evidence specific to children’s therapy services indicates that:

1. Positive care outcomes are related to provision of family-centred services that focus on outcomes related to children’s lives [4,21,22].

2. Capacity to offer appointments to new cases is restricted by therapists allocating time to see children who are already on their caseloads [1,23].

3. Therapists rarely use specific goals to guide allocation of resources [23].

4. Goals, even if present, are rarely shared with the child or parents [8,20].

5. In the absence of shared goals, therapists allocate resources based on their beliefs, values, and emotions (*e.g.*, therapists feel great responsibility for children on their caseloads and guilt for not providing treatment for these children) [20,24].

6. Therapists rarely evaluate the effects of treatments [23].

From this, our hypothesis was that access, equity, and efficiency in children’s therapy services may be improved by optimising clinicians’ resource use; specifically, by supporting clinicians to focus more on treatment goals that are jointly agreed with the child and the family. We propose that efficiency in clinicians’ practice and, through this, access at service level, can be improved through clinicians’ performance of three ‘target behaviours’ [7]:

1. Identify clear and specific treatment goals that are important to the child and family (hypothesis: such goals direct therapists to only take actions that are most likely to contribute to meaningful and effective treatment outcomes for the child and the family).

2. Agree the treatment goals with the child, parent, and/or educational staff (hypothesis: agreed treatment goals encourage mutual commitment to and engagement with the treatment activities). ‘Agree the treatment goals’ is used here in an everyday sense of the phrase, to mean that the therapist discusses the goals with the child, parent, and/or educational staff in such a way that a mutual agreement is reached.

3. Evaluate the child’s progress towards the goals (hypothesis: feedback about the effects of the treatment provides information to the clinician, the child, and the family about when to stop or change treatment).

Rehabilitation clinicians find it difficult to identify and agree shared goals with patients [25-27], and, to date, there have been no evidence-based interventions to implement shared goal setting in children’s clinicians’ practice. A previous study used the MRC complex interventions framework [28,29] to systematically develop an intervention (titled ‘Good Goals’) to encourage implementation of the three target behaviours in the context of children’s therapy [7].

As the first formal study to evaluate the Good Goals intervention in practice, the present study investigated the use of Good Goals in one children’s therapy context, specifically children’s occupational therapy. The specific objectives were to: (1) identify factors related (qualitatively and/or statistically) to the uptake and adoption of the Good Goals intervention; (2) investigate perceived changes in service delivery and actual changes in therapists’ goal setting during the uptake and early adoption of Good Goals; and (3) evaluate the cost of delivering and adopting Good Goals. These objectives correspond to the MRC Framework for developing and evaluating complex interventions [29], specifically to the aspects concerning ‘modelling processes and outcomes’ and ‘feasibility’.

## Methods

**Figure 1:**
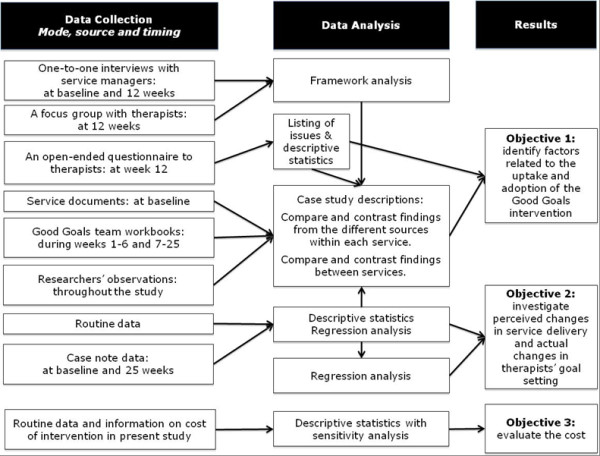
Overview of the methods and the research process in the study.

Three prospective mixed methods case studies [30] (where a ‘case’ was a service, consisting of therapists within the service and children on the therapists’ caseloads) were conducted (see Figure 1 for an overview of the methods and the process). The Good Goals intervention was delivered over 25 weeks (see below). Data were collected before, during, and after the intervention delivery about: (i) the contexts of adoption; (ii) service managers’ and therapists’ perceptions about changes in practice during the adoption; (iii) actual changes in therapists’ actions and in children’s length of time (LoT) on caseloads; and (iv) the cost of intervention delivery and adoption. The study had National Health Services (NHS) Research Ethics Committee approval (No 08/S0801/84).

## Sampling and recruitment

The aim was to recruit a cluster sample of three services, 30 therapists, and 240 children’s case notes. The services were purposively selected to include a spread of services that were ‘keen’ and ‘reluctant’ to participate; to include variation on a range of contextual (*e.g.*, geographical, economic and organisational) settings; to cover children with a range of clinical conditions and of various ages as typically seen in clinical practice; and to include therapists with different levels of experience. NHS-based children’s occupational therapy services in Scotland routinely see children in a variety of settings (schools, clinics, and at homes), and previous research has shown that variation in service delivery approach ( *e.g.*, intensity of intervention provided, approaches to interventions) varies as much between individuals within a service as between services [20,23]; thus, these were not used as sampling criteria.

All therapists within a service were recruited (an agreement by each therapist to participate was a criterion for inclusion), and informed consent was taken from therapists and managers. Parents of children whose case notes were sampled for inclusion were informed of the study and were provided an opportunity to opt out.

## Intervention

Good Goals is a multifaceted intervention built on the assumption that changes at the service, therapist, and child levels are interlinked in that services consist of therapists whose caseloads, in turn, consist of children. Good Goals has been systematically developed based on theory, evidence, and input from NHS therapists [7]. Good Goals is here described in terms of its content, mechanisms of change, and delivery. These components are recommended for describing interventions to change clinicians’ practice [31]:

1. Content: Good Goals consists of eight behaviour change techniques (‘goal specified’, ‘graded tasks’, ‘rehearsal’, ‘social processes of encouragement, support, and pressure’, ‘demonstration by others/modelling’, ‘self-monitoring’, ‘feedback’, and ‘contract’).

2. Mechanisms of change: The eight techniques are targeted at therapists’ beliefs, skills, and behavioural regulation, residing within seven theoretical construct domains that are proposed to be pathways to change in clinicians’ actions [32] and that are hypothesised to determine therapists’ performance of the three target actions [33].

3. Delivery: Good Goals is delivered by a trained facilitator (using standardised intervention materials) through three modes of delivery (two group-based training sessions, tools for facilitating change, and two team workbooks). It is delivered at therapists’ place of work. In order to maximise its acceptability [7] and effectiveness [12,34]. Good Goals is delivered at the level of a whole service (and teams within it), rather than individual therapists.

A detailed description of the development of Good Goals (content, delivery, and intensity and frequency for each component) is provided in a parallel methodology paper.

## Data collection materials and procedures

### Interviews and a focus group

Interviews with service managers (n = 4) at baseline and at 12 weeks into the intervention, and a focus group with a sub-sample of therapists (n = 8), also at 12 weeks, were used to investigate participants’ perceptions of delivery and adoption of Good Goals. The topic guides were structured around the seven theoretical construct domains targeted by the intervention (for full details, please see Additional file 1: Appendix).

### Questionnaires

A brief open-ended questionnaire was distributed to all participants at 12 weeks to explore the perceived advantages/disadvantages and facilitators/barriers related to the intervention.

### Routine data and service documents

Data about the demographics of the populations covered by each service were obtained from the General Register Office for Scotland, Information Services Division (ISD) Scotland, and the Office for National Statistics. Data about each service were collected from services’ documents (*e.g.*, operating manuals, the standard paperwork used by the service, and any service/organisational policies) and the services’ monthly statistics ( *e.g.*, numbers of referrals received, new children seen, children discharged). The nature and format of these data varied from service to service; all available data for each service were collected.

### Workbooks and researchers’ observations

Data related to intervention uptake were collected from intervention workbooks completed at team level (workbooks were one of the modes of delivery for the behaviour change techniques included in the intervention—see ‘Intervention’ above—and provided data on the frequency of the meetings, the number of therapists attending, and summaries of the contents of discussions); from the monthly support calls to service managers; and through researchers’ general observations during data collection (recorded as field notes), intervention delivery, and feedback.

### Case notes

Data for calculating LoT (*i.e.*, date when child was first seen by the service and date when child was discharged), therapists’ performance of the target behaviours and children’s characteristics (age in months, diagnostic category) were extracted from current case notes from all therapists in the participating services at baseline and at 25 weeks. To collect these data, a pre-specified data extraction form was used. This included explicit guidance notes for deciding how information in the notes should be coded. Both the form and the guidance notes had been previously used by the research group in a similar study. The data extraction form and guidance notes are available from the first author.

### Data analysis

The overall approach was: data from each source were initially managed and analysed separately (see below); and the synthesis focused on complementing (*i.e.*, enhancing, illustrating and clarifying) findings from one source with findings from another and on expanding ( *i.e.*, widening) the breadth and range of inquiry by drawing on one source of data to follow up and extend findings from another [35]. Specific methods to analyse the different types of data are described below.

### Qualitative analysis

The interviews and focus group were recorded and transcribed, and the transcripts analysed using the framework approach [36]. The framework consisted of an elaborated version of the theoretical construct domains [37]. NK and SBR independently coded each transcript, discussed the themes that emerged, and agreed codes for the themes; EASD and EMD critiqued the framework and the codes (for full details, please see Additional file 1: Appendix). Open-ended data from the questionnaires were transcribed into Microsoft Office Excel to identify frequently mentioned issues.

Findings from the framework analysis for each service were compared and contrasted with the routine data, service documents, workbooks, and the researchers’ observations using a case study approach [30]. The findings were then compared and contrasted between services, and with the findings from the questionnaires.

### Quantitative analysis

Data about the target behaviours and from routine sources were summarised using descriptive statistics. LoT was calculated by using the ‘date first seen’ and ‘date discharged.’ An estimate of the intervention effect on LoT was obtained from a linear regression model comparing pre- to post-intervention LoT, adjusted for the age and diagnosis of the child and the clustering effect [38] of the therapist. Bootstrapped 95% confidence intervals around this estimate were derived due to the skewed nature of LoT [39]. Estimates of the effect of the intervention on the behaviour data extracted from case notes were analysed in a similar fashion using logistic regression, with estimates presented as odds ratios and 95% confidence intervals. The statistical analyses were conducted using Stata 11 [40].

### Costs for intervention delivery and adoption

The mean cost of receiving the intervention per service was calculated based on staff costs (facilitator, therapist, and secretary time, as well as facilitator accommodation), travel costs (for facilitator and therapists) and consumables (handouts, workbooks). The costs were derived from routine sources and information about expenditure in the present study. Data analysis was based on three-point estimates of service size: eight therapists (small service), 19 therapists (mid-sized) and 26 therapists (large). It was assumed that each service included a service manager, and that for a large service two intervention facilitators would be required. Sensitivity analysis was conducted to test the sensitivity of the results assuming higher and lower staff costs.

## Results

Four services were approached. Three services (n = 46 therapists and their 558 cases) participated (see below). One service manager declined, stating waiting list and caseload pressures as the reasons for non-participation.

The results are reported in four sections: the participating services and their key attributes (as the specific health service contexts may have influenced the intervention delivery, uptake, and adoption); the factors related to the intervention uptake and adoption; changes in service delivery and the study outcomes during the study period; and the costs of adopting the intervention.

### The participating services

The three participating services had different attribute profiles. Services A and B had more senior therapists than Service C. Services A and B covered urban, town, and rural settlements, while Service C covered solely urban areas. Services B and C covered areas of significantly low and high deprivation, respectively (Table 1).

**Table 1:** Characteristics, at baseline, of the therapists; the geographical locations and populations covered by the services; and the children on the services’ caseloads

			**Service A**	**Service B**	**Service C**
			**n**^¥^**= 25**	**n**^¥^**= 17**	**n**^¥^**= 5**
**Therapists’ characteristics**	Band n(%)	4:	4 (16%)	0 (0%)	1 (20%)
		5:	0 (0%)	0 (0%)	0 (0%)
		6:	5 (20%)	5^a^ (31%)	3 (60%)
		7:	15 (60%)	10 (63%)	0 (0%)
		8:	1 (4%)	1 (6%)	1 (20%)
	Years as therapist (Median[IQR])		13 (8-17)	15 (9-26)	7 (4-12)
	Years in paediatrics (Median[IQR])		8 (2-14)	7 (3-16)	6 (3-12)
**Geographical and population characteristics**	Miles required to travel to attend weekly Good Goals meetings with colleagues (mean[SD])^b^		7.0 [8.7]	15.2 [13.6]	0
	Age of the children on caseload at baseline in years and months (mean [SD])		5y 7m	4y 5m	6y 4m
			[3yr 11m]	[3yr 4m]	[3yr 10m]
	% of area in most deprived 15% in Scotland [53]		13.3	4.8	29.4
**Medical diagnoses of the children on caseloads (%)(ordered based on level of medical complexity from high to low)**	Cerebral Palsy		23	41	18
	Other (*e.g.*, global developmental delay, muscular dystrophy)		55	45	41
	Autistic spectrum disorder/ Attention deficit hyperactivity disorder/ Tourette’s syndrome		14	9	27
	Developmental coordination disorder/dyspraxia		2	7	0
	No medical diagnosis		20	16	29

^¥^n here refers to number of therapists in the service (demographic and case note data was collected from all therapists in each service). ^a^ Data are missing for one senior therapist. ^b^ Travel distances calculated using Google maps (http://maps.google.co.uk/).

While all of the services provided mainly community and outpatient care, services A and B also had some inpatients. Services A and B covered entire Health Boards while Service C covered a Community Health and Care Partnership. All the service managers described the remit of their service similarly, the essence of which is captured in the mission statement for Service A: ‘To enable children and young people to meet their highest potential in everyday life.’

The services differed in their structure and processes related to management of patient flow. Service A consisted of three clinical-speciality teams (based on diagnostic groupings) and one ‘generic’ team (Table 2). The service manager oversaw acceptance of referrals to the service, and allocated children to the teams. In the past, each team had had its own identity, norms, and caseload management processes (*e.g.*, ways of assessing, setting goals, and reporting), and both the manager and the therapists reported that the teams continued to have limited interaction between them:.

"‘…[the teams] were very much working as [separate] services… They had their own folders with their policies and procedures… [and although things have improved] we’ve still got a long way to go, and when things pop up people tend to go back to their own teams.’ (Manager, Service A)"

**Table 2:** The structure, demand and resources for each of the participating services

**STRUCTURE**		**DEMAND**			**RESOURCE**	
		**Referrals in the past 3 months**^**a**^**(n/month)**	**Children waiting(n)**	**Children on caseload(n)**	**Staff(WTE)**^**b**^	**Children per WTE staff**
**SERVICE A**		**24/28/33**	**93**	**545**	**18.2**	**42.0**
Generic		8/10/12	38	181	5.75	38.1
Coordination difficulties		11/15/12	49	127	3.0	58.7
Physical disabilities		4/1/6	6	146	6.45	23.6
Mental health		1/2/3	Not available	Not available	3.0	Not available
**SERVICE B**		**Not available**	**123**	**344**	**11.41**	**40.9**
Coordination difficulties		26/21/13	109	91	3.0	57.0
Physical Disabilities	Special schools			28	1.0	
	Pre-school	Not available	12	46	2.367	26.6
	Team 1	Not available	2	35	1.0	
	Team 2	Not available	0	39	1.0	
	Team 3	Not available	0	58	1.487	
	Team 4	Not available	0	47	1.56	
**SERVICE C**		**12/17/11**	**42**	**186**	**4.64**	**49.1**

^a^Calculated from the three months prior to data collection.

^b^WTE = Whole Time Equivalent 37½ hours per week

Service B had the lowest children-to-therapist ratio (Table 2). It was structured around: four child development teams (CDTs); two school teams; and an outpatient service (Table 2). The CDTs and the school teams saw only children with ‘complex disabilities’ and had no waiting lists. Children who did not meet the criteria to become a ‘team child’ were placed on the outpatient waiting list (Table 2). Referrals were accepted by individual therapists; the manager reported limited control over allocation of children to the teams:

"‘…if we’ve got a child that we’ve seen [at the outpatient clinic] and we think… the team should pick them up; they may not agree with that request.’ (Manager, Service B)"

The service had an operational policy for caseload management and the manager described peer pressure for everyone to adhere to it:

"‘…[the policy is] for thinking through what you would be expected to do [at assessment, treatment, and discharge]. …There is a lot of peer pressure… If somebody finds out that somebody is deviating (laugh)… they would be challenged…’ (Manager, Service B)"

However, therapists described differing motivations to adhere to the policy. Some therapists in CDTs described accepting referrals for ‘team children’ only; they reported a belief that accepting other referrals could result in increased pressure on them. Other therapists felt that accepting only ‘team children’ was de-skilling them; these therapists described a practice of discreetly taking non-team children on their caseloads.

Service C had the highest children-to-therapist ratio (Table 2). All referrals to the service were discussed in a multidisciplinary team meeting, attended by the service manager. While therapists had clinical special interests, all therapists had a responsibility to the overall service provision.

Managers for services A and C described themselves enacting leadership roles, both in general and in relation to Good Goals:

"‘[My role in general is] to have the overall plan and to gain advice and ideas from the team; make a plan and delegate who’s going to do what’ [Manager, service C]"

"‘I have said that we’re signed up to [piloting Good Goals] so therefore they will get the time and that I see this as a priority… [it is] my job to have that longer vision and take them with me.’ [Manager, service A]"

Manager for service B described her role in terms of managing the therapists and the service policy, personnel procedures, and administrative processes:

"‘…my job is about professional standards… I supervise staff and make sure they are trained, that their workload’s okay, sorting out day-to-day management issues—annual leave, recruitment…’ [Manager, Service B]"

She reported a perception that the uptake of Good Goals was likely to depend largely on individual therapists and external factors, and stressed the external pressures and lack of resources as anticipated barriers.

### Factors related to uptake and adoption

Comparison of the intervention uptake and adoption (see Additional file 2: Appendix for summary descriptions) between the three services indicated that the key factors related to the intervention adoption were the mode of delivery for the Good Goals intervention (underpinned by competing demands on therapists’ time), leadership by service manager and, in some instances, therapists’ perceptions of the children and families.

The mode of delivery for the Good Goals intervention was the single most influential factor in its uptake and adoption. The training sessions were well attended across all services (82% to 100% of therapists attended), and participants were observed to engage with the materials delivered within these. In contrast, for the workbooks and Good Goals weekly meetings, the number of sessions completed (mean = 9, SD = 4, per team) was considerably lower than that intended (25 sessions per team). From therapists’ reports, the main barrier to using the workbooks was unclear instructions. The main barriers to the weekly meetings were reported as lack of time, difficulties in organising meetings when a number of therapists worked part-time, and difficulties in travelling to meeting locations.

The weekly meetings were the most commonly reported challenge in adopting Good Goals (reported by 14/17 respondents in Service A; 6/7 in Service B; and 2/2 in Service C). The change techniques delivered during the weekly meetings (especially social support, encouragement and peer pressure; and modelling/demonstration of the target behaviours by others) were reported as the most important intervention ingredients:

"‘…unless you’re coming together it’s not going to achieve its aims and you could quite easily go and do your own thing the way you’ve always done it… It’s definitely about the coming together…’ (Focus Group Service A OT4)"

However, due to the reported difficulties in organising the meetings, there was an ongoing tension between the importance of holding the meetings in order to achieve sustainable change and a threat that the meetings themselves might not be sustainable:

"‘These weekly meetings… if [they] fall by the wayside, I think the quality of what the whole thing is about will go down…’ (Focus Group Service A OT5)"

In terms of the service attributes and adoption, in Service A the service manager’s actions (*e.g.*, providing staff with time to implement change; actively providing encouragement and positive feedback; and changing service-level processes so that they match with the intervention principles) were reported as important facilitators by the therapists (see Additional file 2: Appendix). In Service B, where the manager reported less of a leadership role than in Services A and B, some therapists explicitly commented on the lack of a service-wide approach and commitment to change (see Additional file 2: Appendix). There was no evidence of other service attributes being directly linked to adoption.

Finally, in the questionnaire data, some therapists (5/17 respondents in Service A; 3/7 in Service B; and 0/2 in Service C) reported difficulties in carrying out the target behaviours with particular families (*e.g.*, parents with whom therapists had difficult interactions) or children ( *e.g.*, with complex conditions or of younger age). However, there was no evidence from the case note data analysis post-intervention that therapists were identifying and agreeing goals or evaluating progress differently due to children's age or complexity of condition. Further analysis of the focus group data and the researcher’s observations indicated that therapists’ expressions about difficulty of carrying out the target behaviours were often linked to that individual therapist’s beliefs and values. For example, the following quote illustrates how one therapist’s perception about difficulty in identifying treatment goals with some children was linked to her belief about the content of acceptable treatment goals:

"‘[some children]…come up with absolutely ridiculous goals. Two little ones, both in wheelchairs, who wanted to play football. …you say ‘you can’t do that… you can maybe get ball skills in a different setting’ but no, this little one wants to play with his brothers…’ (Focus Group Service B OT5)"

### Changes observed in the study outcomes

The changes that service managers and therapists reported related to the adoption of the intervention were similar across all three services. The intervention was reported to improve equity of care through ensuring a shared rationale for decisions by 54% [14/26] of the questionnaire respondents. This was also reflected in the focus group discussion:

"‘It’s made a much more equitable service… it’s really helped us to be doing similar things with patients, which we weren’t doing before.’ (Focus Group Service A OT5)"

It was reported to increase therapists’ clarity on role, resource use, and intervention provision by 42% [11/26] of the questionnaire respondents. This was similarly reflected in the focus group discussion:

"‘I think we’ve changed quite considerably since the introduction of Good Goals … we’re much more goals focused … which then really guides us to what’s important for the child … It used to be a standard battery of assessments regardless of what was wrong with the child and what the child and parent wanted …’ (Focus Group Service A OT4)"

Finally, the intervention was reported to improve therapists’ interactions with families and schools by 38% of the questionnaire respondents. This was reflected in the focus group:

"‘It’s definitely changed the focus and [we are] asking a lot more questions. I think it empowers the kids to make a decision about what it is they want to work on (…)’ (Focus Group Service C OT3)"

During the study period, there was a measurable increase in the target behaviours across the three services (see Table 3). Estimated odds ratios (95% confidence intervals) comparing pre-intervention to post-intervention were: identifying goals, 2.4 (95% CI 1.5 to 3.8); agreeing goals, 3.5 (2.4 to 5.1); evaluating progress, 2.0 (1.1 to 3.5). LoT decreased by two months [95% CI −8 to +4 months] across all sites during the study period, adjusted for clustering at therapist-level and for the child’s diagnoses and age.

**Table 3:** Number and proportion of cases where there was evidence of the performance of the three target behaviours at baseline and follow-up

	**Identify clear, specific and time limited goals**				**Agree goals with clients**^**a**^				**Evaluate progress towards the goals**			
	**Pre**		**Post**		**Pre**		**Post**		**Pre**		**Post**	
	**n**	**%**	**n**	**%**	**n**	**%**	**n**	**%**	**n**	**%**	**n**	**%**
Service A^¥^	51	39	46	41	23	18	40	36	32	24	23	21
Service B^¥^	32	27	74	59	13	11	40	32	14	12	45	36
Service C^¥^	7	21	17	46	6	18	12	32	1	3	9	24
**Total**	**90**	**32**	**137**	**50**	**42**	**15**	**92**	**34**	**47**	**17**	**77**	**28**

^¥^Case note data was obtained from all therapists in the three participating services (total n = 46 therapists). ^a^‘Clients’ is here used to refer to the child, parents and/or educational staff.

In terms of contextual factors, the therapists reported, and the researchers observed, that managerial leadership was important for achieving changes in service-level processes that facilitated sustainable, long-term change (see Additional file 2: Appendix). No patterns emerged between the other service attributes assessed and the changes in the target behaviours. For example, the two services in which the largest increases in the target behaviours were observed had the therapists with most and least experience, the lowest and highest demand-to-resource ratios, and the most extreme geographical and population characteristics.

### Costs related to delivery and adoption of Good Goals

The total cost of delivering and implementing Good Goals was estimated at between £8,206 and £33,027 per service. The cost per therapist trained ranged from £1,003 (small group of 8 therapists) to £1,277 (large group of 26 therapists). The cost was largely dependent upon the size of the service, the salary bands of the service’s occupational therapists, and the number of training sessions. The main cost driver was staff (facilitator and therapists) costs.

## Discussion

Adoption of a service-change intervention (‘Good Goals’) in three children’s occupational therapy services was investigated. Therapists and service managers reported that the intervention had advantages related to equity and efficiency of service delivery and during the 25-week study period therapists’ performance of the target behaviours increased substantially. The mode of intervention delivery and leadership by service manager consistently emerged as important factors related to intervention adoption. Some therapists raised concerns about appropriateness of therapists identifying goals, agreeing goals, and evaluating progress with some children and families; further analysis indicated that these concerns may relate to therapists’ other beliefs and values rather than the actions or the families (for further discussion, see below). The cost of Good Goals ranged from £8,206 per service (for a small service) to £33,027 (for a large service).

The changes in the three target actions observed during the study period were encouraging. The obvious question is whether it was only the recording of the target actions that changed as opposed to the actual doing of them. Without direct parallel observation of the actions, it is impossible to answer this question conclusively; however, the researchers’ observations during the study, the data from manager interviews (see *e.g.*, Additional file 2, case A) and the focus group data (see Results) all indicate that the changes in the recorded target actions reflected changes in actual performance, and that these changes were further reflected in other observable changes (*e.g.*, in therapists’ clinical reasoning). The hypothesis underlying data collection in the present study was that information from case notes is more reliable than self-report; considering the cost of collecting data from case notes versus self-report it would be valuable to evaluate this hypothesis in a future study.

The study corresponded to the ‘modelling process and outcomes’ and ‘feasibility’ aspects of the MRC’s new guidance for developing and evaluating complex interventions [29]. It is not possible to draw summative conclusions about intervention effects, causality, or long-term consequences. A rigorous evaluation within a randomised study with adequate sample size and longer follow-up is required to gain a confident estimate of the intervention’s effects. Such an evaluation should also consider outcomes on children’s health and cost-effectiveness (rather than just costs).

### Implications for practice, policy, and further research

The importance of using goals as part of clinical practice is not a new idea. Establishing an explicit goal is fundamental for achievement of outcomes [41,42] and has been proposed to relate to effectiveness of clinical interventions and patients’ adherence to treatments [43], including children [44]. The present study adds to these existing arguments by illustrating how systematic goal setting may also be important for efficient and equitable service delivery.

Existing evidence indicates that rehabilitation clinicians rarely identify and agree clear goals with their clients [27,45,46], and that many of the barriers to this relate to clinicians’ beliefs about goal setting. Beliefs frequently reported by clinicians include: that some patients’ goals are fundamentally incompatible with the clinicians’ goals and responsibilities [27,45,46]; that some patients are unable to engage in setting goals (*e.g.*, due to lack of knowledge, expertise, ability or family dynamics) [27]; that focusing on treatment goals can threaten the clinician-client relationship (*e.g.*, by forcing clinicians and patients to confront differences in values and opinions) [47]; and that clinicians do not have the skills and capabilities to identify and agree goals [20,48]. The present study provided some evidence about the potential of the Good Goals intervention to address these beliefs and thus to support teams to implement patient-centred goal setting in practice.

The adoption of Good Goals was characterised by a tension between adopting all of its components in order to achieve change and the challenges related to the adoption of some of these components. Previous research [49] has shown that while clinicians are more likely to adhere to a change intervention that is compatible with their existing values and practices, it is precisely in challenging these values and practices that the greatest changes in practice are observed. In the development of Good Goals [7], it was acknowledged that some of the techniques delivered (specifically, feedback and self-monitoring) were likely to challenge clinicians’ existing values and were likely to be received with resistance. However, as feedback and self-monitoring were hypothesised to be the most important components for achieving change, it was considered important to include them in the intervention. To reduce the resistance, these techniques were chosen to be delivered together with a technique that was hypothesised to be positively received (specifically, social processes of support and encouragement delivered through weekly team meetings) [7]. The tensions related to adoption of weekly meetings in the present study are therefore consistent with the hypotheses made during the development of Good Goals, and even in its future development it may be not be possible to eliminate them entirely.

## Conclusions

Inefficiency, inequity, and waiting times are problems not just in children’s services but in community-based health and social care more widely [12]. To date, there has been a lack of a systematic, evidence- and theory-based approach that services could adopt to address these problems [12]. The Good Goals intervention is a promising quality improvement intervention that can be delivered and adopted in practice and is perceived by staff to have advantages. Further research is required to evaluate its impact on patient outcomes, effectiveness, cost-effectiveness, and transferability to other clinical specialities/professional groups. If found effective, Good Goals has the potential to improve efficiency and equity of community-based services [40,50-52].

## Competing interests

The authors declare that they have no competing interests.

## Authors’ contribution

NK led the design, conduct and write-up of the project, as well as the qualitative analysis and the overall synthesis. GM and LT led the quantitative and costing analyses, respectively. EMD, EASD, and SBR collected data and made a major contribution to data analysis. LM and JJF made a major contribution to the design. SBR, EMD, and NK co-wrote the first draft of the manuscript; NK led the writing of the subsequent drafts, to which all authors made substantial contributions. All authors read and approved the final manuscript.

## Funding

The study was funded by Chief Scientist Office of the Scottish Government Health Directorates (ref: CZG/3/21). The views expressed in this paper are those of the authors. The funder was not involved in the conduct of the study or preparation of the manuscript.

## Supplementary Material

Additional file 1 **Appendix.** The Theoretical Domains Framework: reflection on its use in the Good Goals mixed methods study [last updated in October 2011]. Click here for file

Additional file 2 **Appendix.** Case studies of Services A, B and C. Click here for file
