# Supplementary material for: Using shared goal setting to improve access and equity: a mixed methods study of the Good Goals intervention in children’s occupational therapy
Source: Implement Sci. 2012 Aug 16;7:76. doi: 10.1186/1748-5908-7-76 (PMC3444894; doi:10.1186/1748-5908-7-76)
Supplement: Additional file 1 — Appendix. The Theoretical Domains Framework: reflection on its use in the Good Goals mixed methods study [last updated in October 2011]. [file 1748-5908-7-76-S1.doc]

**Appendix 1: The Theoretical Domains Framework: reflection on its use in the Good Goals mixed methods study** [last updated in October 2011]

*Authors: Kolehmainen N, Duncan EM, Ryan SB, Duncan EAS, Francis J, McKee L*

There is a range of conceptual frameworks and theories concerning interventions to change healthcare delivery that may be informative for researchers.^1-3^ The Theoretical Domains Framework [TDF]^4^ is one such framework. The TDF was purposively designed to be useful for multidisciplinary teams investigating issues at multiple levels of healthcare delivery.^4^ It was developed by a multidisciplinary team (including psychologists, sociologists, clinicians, health services researchers, statisticians and a public health specialist); was designed to communicate to an interdisciplinary audience, and was intended to cover both individual-level and organisational-level concepts.^4^

The TDF was considered an appropriate conceptual framework for the Good Goals study since: (i) the TDF’s ‘construct domains’ had already been shown to be relevant to the topic area;^5,6^ (ii) the TDF had been designed to facilitate multi-level, multidisciplinary investigations such as the study in question; and (iii) three of the research team members already had experience of using the TDF in previous studies.

This appendix describes and reflects on the use of the TDF in the Good Goals study; specifically on (i) the development of a TDF-based topic guide for the study; (ii) the use of that topic guide in interviews and a focus group; and (iii) the use of the TDF as the framework for analysing the transcripts from the interviews and the focus group.

**Development and use of a TDF-based topic guide in the study**

A study-specific topic guide, based upon the TDF, was developed for use in the interviews with managers and a focus group with therapists. Development consisted of five steps:

1. Selecting domains relevant for the study on the basis of previous literature on occupational therapists’ caseload management.^5,6^
2. Using two existing TDF interview topic guides^4,7^ to develop a topic guide that covered the domains included in the present study.
3. Further adapting the topic guide to ensure its suitability for eliciting issues at team-, service- and individual-levels; this involved a researcher occupational therapist (NK), a health psychologist (EMD) and an organisational researcher (LM) working together to interrogate the topic guide and make the topics broad and open-ended to encourage discussion of a range of issues.
4. Generating explicit, study-specific summary definitions of the theoretical and conceptual underpinnings related to each of the topics covered (see below, table A).
5. Two researchers who had been involved in developing the TDF ‘back-translated’ the study-specific topics to the TDF domains in order to independently confirm that the topics continued to reflect the content of the TDF domains.

In relation to step 4 above, the original paper describing the TDF^4^ does not provide conceptual summary definitions of the domains (instead, it provides a list of constructs). In the course of the development of the topic guide it became evident that impressions of what each topic was intended to cover differed between the research team members. Some of these differences appeared to relate to different meanings given to words depending on the disciplinary and theoretical background of the researcher. The function of the summary definitions in the present study was thus to provide explicit guidance about the intended coverage of each topic to the researcher collecting the data as well as to reassure researchers from different disciplines that the topics they considered relevant were covered by the topic guide.

**Use of the topic guide in interviews and a focus group**

The interviews with service managers were conducted by a researcher (EMD) with previous experience of the use of the TDF in individual-level studies and with experience of semi-structured interviewing. The focus group was conducted by the same researcher and another researcher (EASD) who was experienced in conducting focus groups. The interviews and the focus group were recorded verbatim and subsequently transcribed.

**Use of the TDF as a framework for analysing data**

The transcripts (interviews: n=8; focus group: n=1) were analysed using framework analysis.^8^ In framework analysis the chosen conceptual framework (here the TDF) is used as an initial ‘coding framework’ which is then expanded to reflect the emerging themes from the particular study (here themes related to adoption of Good Goals). This results in a ‘final coding framework’ that reflects both the initial conceptual framework and the emerging themes from the study.

In the present study, researchers from different disciplinary backgrounds and with different expertise (NK, SBR, EMD, EASD) undertook the data analysis. This was so that multiple professional perspectives could be gained, which allowed for a nuanced analysis and unveiling of any tensions and reconciliations in the coding. It also helped to ensure that the coding framework was adapted to reflect the data rather than making the data ‘fit’ into the conceptual framework. The analysis consisted of four steps:

1. Two researchers [NK and SBR] familiarised themselves with the data by reading through the transcripts.
2. One transcript was individually analysed, with each researcher taking notes of the responses and issues, and ‘sifting and sorting’ these into the coding framework by using the domain descriptions (below, Table A).
3. The researchers met to discuss the emerging issues and themes, and to agree sub-codes and themes that were added to the coding framework.
4. The researchers proceeded to categorise (i.e. ‘index’) the remaining transcripts, one by one, according to the revised coding framework. Meetings to discuss and agree further elaborations and changes to the coding framework took place after each transcript. In addition, two further researchers [EASD and EMD] also coded a transcript each and critiqued the coding framework. This process was repeated until all data were indexed and the final coding framework was agreed between all four coders.

Some challenges were found in the use of the TDF as the coding framework for a multidisciplinary, multi-level data analysis. Two researchers (SBR, EASD) who were not familiar with the theories underpinning the TDF domains had difficulties in accessing the framework and using it effectively to code data. This was in large part related to translating between the terminology of the TDF and the terminology used by the different researchers. For example, the researcher with organisational background was unable to identify what certain labels (e.g. ‘behavioural regulation’) were intended to refer to and what the distinctions between the domains were (e.g. the distinction between ‘environmental context and resources’ and ‘social influences’ where the former could be thought to contain the latter in the form of a social environment). Two researchers with previous experience of the TDF and psychological theories related to professionals’ practice (NK, EMD) were able to use the framework; to discuss their justifications for the codes they had assigned; and to illuminate some of the theoretical underpinnings of the different domains to the rest of the research team. However, at times they too struggled to fully understand the intended content of the different domains, and some of their coding differed from that recommended by a senior researcher (JF) with extensive experience in using both the TDF and many of the theories from which it derives.

It took several iterations to achieve consistent coding between the researchers. The researchers often coded the same excerpts of the data with different degrees of specificity where each researcher’s degree of coding specificity varied according to the content of the data. For example, in coding data about a service manager’s actions and leadership approach, the organisational researcher tended to use a wider range of more specific codes than the individual-level researcher who used only one broad code. Upon discussion of the coding, the researchers attributed both the differing degrees of specificity and the divergence in codes to their differing conceptualisations of the domains and the codes. As a result, lengthy discussions were required to negotiate around maintaining the theoretical integrity of the domains whilst reflecting the data and the multiple perspectives on and interpretations of the text.

The final study-specific coding framework is available from the first author. Of the eight domains included in this study, four (beliefs about consequences; beliefs about capabilities; environmental context and resources; and motivation and goals) exhibited substantial commonalities among the four researchers involved in the coding. For three of these domains, the labels and content in the TDF effectively guided the analysis. For one (motivation and goals), the domain label was translated to a study-specific theme that better reflected the data and specific codes emerging from the data (the revised label in the final coding framework was ‘commitment to adopting the target behaviour, and other priorities’). Three further domains offered opportunity for elaboration in their content; for some of these this was also reflected in their label in the final coding framework. In the course of the analysis, the ‘social/professional role and identity’ domain was translated into a theme labelled ‘the role of the service and those within it’; this theme included the role and identity of the service and the boundaries between services (e.g. occupational therapy service and educational services) as well as the roles of the individuals within the service. The ‘social influences’ domain was elaborated to include relationships between services, and both the individual therapist and leadership/management themes were populated. The ‘behavioural regulation’ domain was translated into a theme labelled ‘processes and practices’ and was populated to explicitly include individual-, team-, service-, organisational- and national-level processes and practices. One domain (‘emotion’) had limited data and thus it was not possible to explore it in depth in the present study.

Table A. TDF domains; the interview topics and the related questions in the present study; and the theoretical/conceptual definitions used as a guide by coders for the topics in the present study

| ***TDF domain^4^*** | ***The interview topic, and the questions used to introduce the topic, in the present study*** | ***Theoretical/conceptual definitions used for the topics in the present study*** |
| --- | --- | --- |
| **Professional role and identity** | **The role of the service; relationships within the service and with others**  *“Perhaps you could start by broadly describing your service and its role, and how things are done in your service?”*  *“How would you describe the role that your service has in managing the needs of the children in this region?”*  *“Could you tell me a little bit more about the relationships within the team, and how might these influence the implementation of Good Goals?”* | Perceptions about belonging to a particular social category or group, adoption of group norms and values, boundaries between groups.  Service manager’s role; their approach to leading and managing the team. |
| **Social influences** |  | Effects of other people, relationships and interactions between people.  Support, advice and expectations of others.  Relationships and roles in the wider context of service delivery. |
| **Behavioural regulation** | **Processes and practices related to caseload management**  *“Could you describe any processes and practices related to caseload management that are currently in place in your service?”*  *“What is your impression about the things that you and your team will need to do to implement Good Goals?”* | Processes, procedures, frameworks and ways of doing things that relate to goals, standards or targets. |
| **Motivation and goals** | **Commitment to adopting Good Goals, and conflicts with other priorities**  *“Could you tell me your thoughts on how important will adopting Good Goals be to your service and how does Good Goals fit with the other priorities and aims of your service?”* | Intentions; commitment to a course of action or behaviour; targets and priorities. |
| **Beliefs about consequences** | **Perceptions about consequences and outcomes**  *“How about advantages or disadvantages of adopting Good Goals for your service and to you – do you think there might be some, and if so what might these be?”* | Perceptions about outcomes; advantages and disadvantages. |
| **Beliefs about capabilities** | **Perceptions about barriers and facilitators to adoption**  *“How easy or difficult do you expect it will be to adopt Good Goals? What factors do you think might facilitate the adoption? What difficulties do you expect to encounter?”* | Perceptions of competence and confidence. |
| **Environmental context and resources** |  | Factors related to the ‘setting’: people, team and service, wider organisational factors, cultural, political, physical and financial factors. |
| **Emotion** | **Feelings about implementation**  *“How do you feel and how do you expect your team to feel about adopting Good Goals?”* | Feelings, affect. |
| **n/a** | **Other issues**  “Are there other things that you would like to say about the forthcoming delivery and adoption of Good Goals?” | n/a |

# References

(1) Greenhalgh T, Robert G, MacFarlane F, Bate P, Kyriakidou O. Diffusion of innovations in service organizations: systematic review and recommendations. Milbank Q 2004;82(4):581-629.

(2) Powell AE, Rushmer RK, Davies HTO. A systematic narrative review of quality improvement models in health care. Edinburgh: Quality Improvement Scotland; 2009.

(3) Davies H, Powell A, Rushmer R. Healthcare professionals' views on clinician engagement in quality improvement: a literature review. 1st ed. London: The Health Foundation; 2007.

(4) Michie S, Johnston M, Abraham C, Lawton R, Parker D, Walker, A. on behalf of the "Psychological Theory" Group. Making psychological theory useful for implementing evidence based practice: a consensus approach. Qual Saf Health Care 2005;14(1):26-33.

(5) Kolehmainen N, Francis J, McKee L, Duncan EA. Beliefs about responsibilities, the aims of therapy, and the structure of the therapy process: a qualitative study of caseload management issues in child health occupational therapy. Child: Care, Health & Dev 2012:116.

(6) Kolehmainen N. Optimising caseload management: developing an intervention in children's occupational therapy. Aberdeen: Health Services Research Unit, University of Aberdeen; 2009.

(7) Francis JJ, Stockton C, Eccles MP, Johnston M, Cuthbertson BH, Grimshaw JM, et al. Evidence-based selection of theories for designing behaviour change interventions: using methods based on theoretical construct domains to understand clinicians' blood transfusion behaviour. Brit J Health Psychol 2009;14(4):625-646.

(8) Richie J, Spencer L. Qualitative data analysis for applied policy research. In: Bryman A, Burgess R, editors. Analysing qualitative data. 1st ed. Oxford: Blackwell Publications; 1994. p. 173-194.
