# Supplementary material for: Using shared goal setting to improve access and equity: a mixed methods study of the Good Goals intervention in children’s occupational therapy
Source: Implement Sci. 2012 Aug 16;7:76. doi: 10.1186/1748-5908-7-76 (PMC3444894; doi:10.1186/1748-5908-7-76)
Supplement: Additional file 2 — Appendix. Case studies of Services A, B and C. [file 1748-5908-7-76-S2.doc]

**Additional File 2. Case studies of Services A, B and C.**

**Case study of Service A: *Specialist teams adopting the Good Goals principles with co-ordination by the manager***

In Service A, the findings from the different data sources converged in relation to some of the key themes that emerged. An overall impression was that, during the study, Service A focused on achieving increased consistency of caseload management across the service. The manager reported that adopting Good Goals: exposed differences between therapists and teams in both their beliefs and their practice; and provided them with a structure for seeking a more shared understanding of factors related to caseload management.

“…we’ve had two very different camps… One is very much [sensory integration[[1]](#footnote-2)] and the others are more functional[[2]](#footnote-3) …and never in the middle did they meet... Good Goals has been a really good facilitator in having these difficult discussions about their practice.”

The therapists echoed this, and further emphasised the importance of increased communication between the teams.

“We work quite separately and doing this has made us come together more in meetings by looking at each other’s goals, and challenging that in a fairly positive way.” (OT23 Mental Health Team)

“It’s made a much more equitable service because we’ve all come to our team from different backgrounds… and it’s really helped us to be doing similar things with patients, which we weren’t doing before.” (OT5 Physical Disabilities Team)

The findings indicated that the manager took an active role to facilitate changes both within and between teams. She reported having carried out a range of actions, including providing staff with time to implement change; facilitating discussions; actively providing support, encouragement and positive feedback; and ensuring support from other managers.

“It was about making sure that the staff had protected time… and giving them lots of positive feedback on actually how well they’re doing… talking about it to my manager colleagues to make sure that they know what we’re doing…” (Manager, Service A)

These actions were also perceived as important by the therapists.

“…if we’d not all been together with our manager at the beginning, it might have fallen by the wayside..” (OT5 Physical Disabilities Team)

An overall impression from the data was that Service A had actively integrated the principles from Good Goals into their service delivery. For example, the principles were described to guide the service’s approach to new initiatives such as a diagnostic pathway for children with Autistic Spectrum Disorders. The service manager reported a perception that Good Goals had influenced therapists’ thinking and behaviours to the point where the effects could not be undone.

“It’s like a journey and we can’t go back because our mindsets have changed… the ways the OTs are thinking, the way they’re setting goals, the way that they’re prioritising has evolved and I don’t think you can undo any of that… I think it is becoming embedded in normal practice. ...I don’t think we can [stop it] even if we wanted to, which I think is really positive.”

The case note data analysis indicated that, over the study period, in Service A there had been an increase in therapists’ behaviour of agreeing goals with clients (Table 4). Changes in the other two target behaviours had been smaller. The observations during the data collection indicated that therapists in service 1 had focused on increasing the quality of their goals (e.g. there were examples of high quality goals in the case notes) but that the behaviours had not yet been generalised across cases.

Case study of Service B: *Individuals changing their practice within a dispersed structure*

In Service B, the data from the service manager was limited (she attributed this to being on leave for weeks 5-10 of the study), and the views expressed by therapists diverged both from each other and from the views expressed by the manager. As a result, there was no one uniform narrative of the delivery and adoption of Good Goals at Service B. A related impression from the data analysis was that the adoption of Good Goals in Service B was characterised by a lack of a united, service-wide approach to caseload management. A therapist from the service alluded to this when discussing the differences across the service and the limited accountability of individual therapists to service-level activities.

“…although we’re meeting… but we know that other groups haven’t met and although they do discuss it, I bet none of them have got any [good quality] goals in their notes and you know, who’s gonna take them on?” (OT5, Service 2)

The service manager reported that Good Goals had increased their awareness of some unhelpful caseload management processes and practices.

“I think the way we process patients through the system… [Good Goals] kind of flags up ‘why are we coming back for a review because what’s the goal?’ …it’s flagged up how unhelpful that process actually is.” [Manager, Service B]

However, there was limited evidence of change in these practices and any changes that were reported were driven by individual therapists in relation to their own practice. There was also evidence that some therapists were recording goals in notes retrospectively (i.e. several weeks after starting treatment sessions). Together, these observations suggest that Service B may have been more focused on improving the setting and recording goals than the service-wide caseload management.

The researcher also noted that at the second face-to-face training session some of the more vocal therapists expressed strong negative emotions about the target behaviours and questioned the meaningfulness of the Good Goals principles. The concerns expressed were similar to those identified in previous qualitative studies of occupational therapists’ beliefs about caseload management,[20] and related largely to perceptions about professional role and identity (e.g. beliefs about duty to provide treatment to the child regardless of whether the intervention has effect) and aims of therapy (e.g. conflicting values and preferences between the therapist and a parent or child). An example of the latter is captured below.

“[some children]…come up with absolutely ridiculous goals. Two little ones, both in wheelchairs, who wanted to play football. …you say ‘you can’t do that… you can maybe get ball skills in a different setting’ but no, this little one wants to play with his brothers…” (OT5, Service B)

At follow-up case note data collection, the informal comments to the researcher indicated that therapists’ views and experience of Good Goals continued to vary from “very positive and actively using goals” to “uncertain about how to set goals to” to “I don’t think it is very useful”. The case note data analysis indicated that, over the study period, there had been a substantial increase in therapists’ performance of all three target behaviours (Table 4).

**Case study of Service C*: A united team driving for change in processes***

In Service C, the views expressed by the manager and the therapists, and the observations made by the researcher, converged and, similarly to Service A, there was a uniform narrative about the adoption of Good Goals. This was despite the manager in Service C being on leave for part of the study (weeks 14-25).

An impression from the interviews and researcher’s (NK) observations at baseline had been that, before Good Goals, Service C had placed a strong emphasis on a ‘self-management’ approach (i.e. the importance of parents’ ability to support the child independently of the therapists). For example, the service had implemented several initiatives to facilitate this, including parent-child workshops for children with motor co-ordination difficulties and screening appointments for children with Autistic Spectrum Disorder. However, before Good Goals the self-management approach had not been successful with ‘complex learners’ (i.e. children with more ‘complex’ conditions/life situations).

“We’ve got a drawer full with big massive thick files; they’re our complex learners. …if you do try and discharge them the doctor doesn’t like it, the parent doesn’t like it, the school doesn’t like it.” (Manager, Service C)

Over the course of the study, Service C focused on changing caseload management processes. One of the key developments was a structured approach to appointments with the ‘complex learners’. The approach aimed to empower parents and to change other professionals’ expectations of the service by guiding the parents and/or the child to identifying specific goals – and strategies to achieve these. This approach also acknowledged that the ‘complex learners’ had been on the service’s caseload for a long time and required support to adapt to the service’s more goal-focused way of working.

“…if we turned around now and said ‘…we’re gonna discharge you’ it would be too much of a shock. …So we’ve agreed we can keep them on but we need to know why. […we send them a letter] before they come for an appointment [to encourage them to identify] ‘what do you want to work on?’ We use the question from Good Goals...” (Manager, Service C)

Therapists in the service perceived that adopting this new structure to appointments had empowered them to change their thinking and practice.

“…it’s been really helpful in changing our thinking.... It’s given us a way to look at discharge for these more complex cases that traditionally would have been on the caseload until they left the service at eighteen” (OT3, Service C)

Another characteristic of the adoption of Good Goals at Service C appeared to be the manager’s high level of awareness of the “ripples” that any changes could cause to other services. This was reflected in the service’s approach to change. Rather than pushing for the change overtly the service manager hoped that others would notice the positive outcomes of change through practice.

“I’ve not been particularly vocal about what we’re doing. What [other members of the multi-disciplinary team] are going to see is that we would be writing very clear reports after we have seen the children...” (Manager, Service C)

The service manager described high levels of within-service support and shared learning.

“…everyone has been very supportive of each other. …nobody feels very confident in doing it …we’re all just learning and sharing...” (Manager, Service C)

At the follow-up case note data collection, the therapists appeared positive about their goal setting, proud of their achievements, and relaxed about the follow-up data collection. The case note data analysis also indicated that, over the study period, there had been a substantial increase in therapists’ performance of all three target behaviours (Table 4).

1. An approach that focuses on sensory processing functions proposed to underlie skills and abilities [↑](#footnote-ref-2)
2. An approach that focuses on execution of skills and abilities in the context of daily activities [↑](#footnote-ref-3)
